# Building diagnostic systems in Sierra Leone: The role of point-of-care devices in laboratory strengthening

**DOI:** 10.4102/ajlm.v9i2.1029

**Published:** 2020-04-01

**Authors:** Rashid Ansumana, Fatmata Bah, Kan Biao, Doris Harding, Mohamed B. Jalloh, Ann H. Kelly, Francess Koker, Zikan Koroma, Mambu Momoh, Mohamed H. Rogers, James Rogers, Alice Street, Eva Vernooij, Isatta Wurie

**Affiliations:** 1 School of Community Health Sciences, Njala University, Bo, Sierra Leone; 2 Kings Sierra Leone Partnership, King’s Centre for Global Health and Health Partnerships, Freetown, Sierra Leone; 3 Sierra Leone-China Friendship Biological Safety Laboratory, Chinese Center for Disease Control and Prevention, Beijing, China; 4 National Institute for Communicable Disease Control and Prevention, Beijing, China; 5 Public Health Laboratories, Sierra Leone Ministry of Health and Sanitation, Freetown, Sierra Leone; 6 Department of Community Health, Faculty of Clinical Sciences, College of Medicine and Allied Health Sciences, University of Sierra Leone, Freetown, Sierra Leone; 7 Global Health and Social Medicine, Kings College London, London, United Kingdom; 8 Clinical Laboratories and National Coordinator BioBanking and Biosecurity, Sierra Leone Ministry of Health and Sanitation, Freetown, Sierra Leone; 9 Kenema Government Hospital, Viral Hemorrhagic Fever Consortium, Kenema, Sierra Leone; 10 School of Nursing and Medical Laboratory Sciences, Eastern Polytechnic, Kenema, Sierra Leone; 11 College of Medicine and Allied Health Sciences, University of Sierra Leone, Freetown, Sierra Leone; 12 Laboratory Technical Working Group, Sierra Leone Ministry of Health and Sanitation, Freetown, Sierra Leone; 13 School of Social and Political Sciences, University of Edinburgh, Edinburgh, United Kingdom

The 2014–2016 Ebola Virus Disease (EVD) outbreak highlighted the vital importance of investing in West Africa’s laboratory infrastructure and systems. In the absence of facilities capable of handling highly infectious pathogens, the national response across the region was hamstrung by costly delays in case identification and blind spots in epidemiological surveillance. The rapid development of EVD diagnostic tools that could speed up testing and be used at or near the point of care became a public health priority. To expedite their deployment, a series of cross-sectional research and development initiatives were launched including innovative financing mechanisms, data-sharing platforms, public-private partnerships and accelerated regulatory pathways.^1^

A number of novel EVD diagnostics were trialled in Sierra Leone.^2,3,4,5,6,7^ Guided by a national testing algorithm, rapid diagnostic tests (RDTs) such as ReEBOV (Corgenix, Broomfield, Colorado, United States) and the OraQuick (Orasure Technologies, Bethlehem, Pennsylvania, United States), intended for use at the primary care level, and automated real-time reverse transcription polymerase chain reaction (RT-PCR), primarily run on the benchtop, underwent rapid validation and performance evaluation through the World Health Organization’s Emergency Use and Assessment Listing mechanism.^8,9,10,11^ The ReEBOV test was temporarily deployed in a field setting in Sierra Leone and another rapid test, the DSTL EVD Rapid Diagnostic Antigen Test, developed by the Defence Science Technology Laboratory in the United Kingdom, was validated in hospital and primary care settings.^6^ However, biosafety concerns, related to challenges in procuring, distributing and providing training for the use of personal protective equipment to safely draw, handle and dispose of blood samples suspected of being infected with Ebola virus, in addition to imperfect sensitivity and specificity, meant RDTs were rarely used outside the laboratory. Instead, they were deployed at laboratories in key areas of the country alongside automated RT-PCR machines, such as GeneXpert (Cepheid, AB, Sunnyvale, California, United States) and BioFire Film Array (BioFire Diagnostics, Salt Lake City, Utah, United States), that could quickly confirm their results. In addition to commercially available platforms, international public health agencies and non-governmental organisations brought their own in-house assays, some of which came to provide a point of reference for field evaluations of novel tests.^12^ Collectively, these advances in rapid and proximate point-of-care diagnostic capacity helped bring the outbreak to an end and became the cornerstone of Sierra Leone’s enhanced surveillance plan, intended to sustain a ‘resilient zero’ number of cases.^13^

In the aftermath of Ebola, some of these devices and machines were left in clinical and surveillance laboratories as part of the post-Ebola effort to improve disease detection and response capabilities. An RT-PCR machine in the biosafety level 3 laboratory supported by the China Center for Disease Control and Prevention (China CDC) currently serves the Freetown area, while a GeneXpert machine for point-of-care testing supports upcountry EVD surveillance in the Bo and Makeni Government Laboratories. While important for future outbreak response, the availability of EVD diagnostics is clearly only a minor part of public health emergency resilience.^14^ The handover of epidemic preparedness from international partners to national institutions offers a unique opportunity to think through the broader, system-level needs for the accurate and rapid diagnosis of infectious diseases. Critically, more attention is needed to grasp how national strategic plans for strengthening laboratory infrastructure can be best adapted to and advanced by the 2024 Global Health Security Agenda.^15^

Global health and policy debates, largely dominated by institutions in Europe and the United States, have focused on the development of new diagnostic technologies appropriate for resource-poor settings, which generally means that tests should be affordable, easy to use, rapid, and available at the point of care.^16,17,18^ Less prominent in these discussions are the voices of experts in laboratory medicine from the countries for which the rapid tests are designed. The experience of Sierra Leonean laboratory medicine experts, both during and after the outbreak, offers a valuable, under-recognised and much-needed perspective on the role of point-of-care tests and diagnostic innovation in global health.

On 04 March 2019, a multidisciplinary group of policymakers, biomedical and social scientists and local experts in the field of laboratory medicine convened to discuss the role of point-of-care diagnostic devices in outbreaks and their integration with healthcare infrastructure in Sierra Leone. The meeting was organised by the DiaDev research project, funded by the European Research Council under the Horizon 2020 Framework, which investigates the development of point-of-care diagnostic devices in global health and their role in transforming health systems in resource-constrained settings (www.diadev.eu). The meeting featured short presentations as well as a panel discussion with Sierra Leonean laboratory scientists and health workers, who shared their first-hand experience using rapid diagnostic tests during the Ebola outbreak. Three key insights from the day are highlighted here as disruptive and constructive contributions to debates about diagnostic futures in Africa.

## Diagnostic systems not diagnostics

Point-of-care devices are often championed as solutions for places with weak or no laboratory infrastructure. For EVD alone, there are up to 27 assays at different stages of development or use, including 9 antigen-based rapid tests and 18 RT-PCR assays for EVD.^19^ But the lower sensitivity of point-of-care devices means they are often integrated into complex algorithms that require confirmatory testing prior to treatment, which necessitates a wider public health laboratory network. Moreover, even in the case of tests approved for stand-alone use, routine quality assurance systems require proficiency training and regular cross-checking of samples by a reference laboratory.

Lessons from Sierra Leone show that effective use of point-of-care devices also depends on the existence of regulatory capacity to safeguard the quality of point-of-care devices. Many RDTs on the Sierra Leonean market are of questionable quality and have not passed through the Pharmacy Board, the agency that regulates medicines and diagnostics. Representatives of the Ministry of Health and Sanitation underscored that, while the Central Public Health Reference Laboratory is mandated with overseeing quality assurance and post-market validation of diagnostic tests for specific diseases such as HIV, there is a need to expand regulatory and quality assurance systems for all diagnostics, especially those for the national priority special pathogens. One current priority for the Central Public Health Reference Laboratory is to improve the post-market regulatory control by re-introducing lot verification testing of diagnostic devices before and after they are distributed to clinical settings. Enhancing these systems will curtail the supply of low-standard tests and also empower national clinical and public health systems to demand more rigorously tested and, arguably, superior products from international vendors.

Moreover, further investigation is needed into how these devices are being used in clinical practice and their capacity to improve patient care. For example, in the country’s main referral hospital, rapid tests for hepatitis B, *Helicobacter pylori* and urine analysis, are used inside the clinical laboratory, but routine reporting systems in the laboratory mean results are only given back to clinicians the next day, even when the test result is ready earlier, impacting the ability of point-of-care tests to reduce result turn-around times. The migration of rapid diagnostic technologies into clinical laboratories also poses the risk that technologies designed to provide preliminary clinical diagnosis in places without laboratories actually replace and diminish existing laboratory capacity.

Point-of-care tests do not present an alternative to investment in central laboratories, which remain essential for quality assurance, confirmatory testing and research.^20^ Particularly among patient populations likely to be afflicted by more than one disease, the availability of a comprehensive suite of basic laboratory tests located at a primary care level, as outlined by the World Health Organization’s recently published Essential Diagnostics List,^21^ is critical to the establishment of person-centred healthcare. Rather than asking whether we should invest in laboratory strengthening or point-of-care tests, we need to look at how we can best build diagnostic systems and what role new diagnostic technologies might play in strengthening those systems.

The World Health Organization framework for health systems strengthening has drawn critical attention to the six technical ‘building blocks’ of health systems: service delivery, health workforce, information, medical products, financing, and leadership and governance.^22^ All these areas are fundamental to the operation of a diagnostic system, whether this means training the health workforce in the use of new diagnostic devices, ensuring health information systems are aligned with the data from new diagnostic devices, securing supply systems to ensure reagents and other equipment are at hand, or building quality assurance systems that connect peripheral health facilities to central laboratories. But while the World Health Organization framework focuses on technical elements, our on-the-ground experience of laboratory medicine in Sierra Leone emphasises the relationships between people, technologies and infrastructure that enable diagnostic systems to work. These relationships are social and political, as well as technical, and their understanding requires attention to the interaction between local and global normative frameworks and value systems in addition to formal management structures.^23^ Building on the work of social scientists working in this area, we propose that systems thinking entails a shift away from a focus on diagnostic technologies to understanding the way relationships between people, technologies and infrastructure unfold within everyday diagnostic processes and practices.^24^

## The hidden burdens of technology

Recent global health policy discussions about diagnosis in low- and middle-income countries have focused on the need to incentivise markets for new diagnostic technologies. Point-of-care tests that can be sold as affordable products are often championed as simple and easy-to-use solutions for places with limited infrastructure. But the Sierra Leonean experience suggests that new technologies also place a significant un-costed burden on the health system and the people who work in it, reinforcing the existing findings from social science research that point-of-care tests in Africa have unexpected, intensive infrastructure requirements.^25,26^

Contributors to the workshop noted that the burden is most sharply felt in supply chains, especially around the need for a constant and reliable supply of reagents, and transportation costs for confirmatory testing as part of quality assurance. In a recent measles outbreak in Sierra Leone in 2018, limited resources for specimen transportation to the reference laboratory from districts, provided through the national surveillance programme, and the lack of a full complement of reagents for analysis, hampered timely diagnosis. In this instance, preliminary diagnosis with a point-of-care test may have provided a stopgap for a quicker response. However, the 2012–2013 cholera outbreak in Sierra Leone showed that point-of-care tests often lack the required sensitivity, with only 10% of cases confirmed positive by conventional culture in the bacteriology laboratory, resulting from weak international and national regulatory frameworks safeguarding the quality of cholera RDTs.^27^ Point-of-care tests with very high sensitivity and specificity need to be coupled with confirmatory testing infrastructure and proper coordination between government institutions with oversight roles to ensure that the right types of kits are used for routine diagnosis.

Beyond the compromises in accuracy that point-of-care test designs often entail, every new diagnostic device and each iteration of platforms already in use requires a retraining of health workers and laboratory staff, creating heavy logistical, administrative and financial costs on public health institutions. These expenditures are compounded by those associated with the routine operations of the laboratory, including the purchasing of equipment, reagents, storage of large volumes of tests that require refrigeration, everyday maintenance and, critically, management of non-biodegradable waste, generated by point-of-care tests such as the cartridges used in GeneXpert machines or the test cassettes holding reagent strips for viral hepatitis tests. In the past, these institutional overhead costs have been mainly supported by donors as part of short-term research budgets. To support efforts to improve quality-assured diagnostic operations, a clearer understanding is needed of the hidden costs of diagnostics and where they fit in the broader financial landscape of national laboratory infrastructure and systems.

## What is diagnosis for?

Diagnostic testing is not a medical intervention, but a means of generating information for evidence-based medical practice. The question we must ask is: what can be done with this information in this place, with these resources, by these people? The point of diagnosis is not just to know what disease someone has, but to be able to act on that knowledge – whether in terms of public health measures or therapeutic intervention. As the panellists in a round table discussion dedicated to reflections on the use of point-of-care tests during the EVD outbreak made clear, detecting the presence of a pathogen is just one component in a larger set of testing needs. Diagnosing EVD is key to public health measures, such as the isolation of patients, safe burial of bodies and contact tracing, but for clinicians it is only the starting point for therapeutic intervention. When it comes to care, other tests, such as liver function or electrolytes are arguably more important and depend on a generalised laboratory capacity. For example, point-of-care bedside analysers, such as the i-STAT (Abbott, Lake Forest, Illinois, United States), which can help monitor patients in the red zone of the treatment unit, were crucial to saving lives during the outbreak but received far less attention than Ebola diagnostics. Intermittent power outages can affect the validity of standard laboratory tests, such as blood culture, with dangerous implications for clinical outcomes during routine care practices but also poses a significant challenge for clinicians working during the outbreak.

Finally, as we look ahead towards building a sustainable healthcare laboratory system in Sierra Leone, we need to link the question of diagnostic use to that of diagnostic value, or ‘what worth does a specific diagnostic test have for the particular health system?’ At a cost-effectiveness level, answering this question might mean calculating in the hidden burdens that new technologies generate, including workload burdens involved in using tests and reporting results, burdens on patients to travel for testing, and burdens on the health system to provide training, quality assurance, regulation, procurement and supply, and waste disposal systems. Some point-of-care tests may reduce the cost of care in wealthy countries but are a burden in resource-constrained countries such as Sierra Leone.^28^ For example, point-of-care PCR tests, such as the BioFire Film Array system and GeneXpert machines, require the use of expensive cartridges that deter their routine use for testing; the cost of a BioFire Gastro-intestinal panel ($155.00) is higher than the minimum monthly wage in Sierra Leone.^28^

Calculations of value would caution against the hasty introduction of new, more advanced testing devices for particular diseases, when local capacity for testing already exists. For instance, investing in simple and affordable technology such as ammonia solution for assessment of haemoglobin using a colorimeter may offer better value for money than handheld point-of-care haemoglobin meters with costly cuvettes (e.g. HemoCue, Ängelholm, Sweden), which also require the training of laboratory staff and placement in coordinated systems. As another example, while biosequencing may be a compelling orientation for research on emerging infections, for clinical use in a resource-poor context, its running costs are plainly prohibitive.

The value of a diagnostic test cannot be determined merely by the accuracy of the test or the global health priority of the pathogen but on the basis of local needs and consideration of the test’s clinical and operational benefits. The work that the Viral Hemorrhagic Fever Consortium did in partnering with a local institution in the design and development of an RDT for Lassa fever virus is an example of how local priorities can be built into innovation processes from the outset and such local institutional partnerships are to be encouraged in the development of future diagnostic systems in African countries.^29^ A novel diagnostic test’s value, moreover, is not the same as its value for money. Beyond a bottom-line economic analysis, the adjudication of diagnostic value requires attention to the everyday lives and work of patients, clinicians, nurses, laboratory technicians, surveillance officers and public health officials.

### Conclusion

Point-of-care tests are never introduced in a vacuum. Ebola brought visibility to the need for improved diagnostic systems in low- and middle-income countries, but even countries severely lacking in laboratory infrastructure have pre-existing and highly specific diagnostic needs and capacities. Sierra Leone had a National Medical Laboratory Policy and five-year strategic plan in place when the Ebola outbreak occurred. While the renewed focus on global health security strengthening and, by extension, laboratory system improvement, is welcome, it is critical that the national tools and plans put in place are aligned to any new diagnostic devices or laboratory strengthening initiatives and allow for national priorities to be addressed.

Point-of-care diagnostic devices are often championed for their ability to work anywhere, but technologies are never autonomous from the systems in which they are used. Diagnostic innovation needs to start from existing diagnostic systems and national policies and plans. This requires the involvement of social science research in understanding the local context into which point-of-care testing devices are introduced and in identifying local priorities for strengthening diagnostic systems. Global health research and development should be directed at making diagnostic systems become workable in their own right, rather than finding ways that an ever-increasing range of individual technologies can be best accommodated.

Post-Ebola, Sierra Leone is focused on improving the resilience of healthcare delivery.^30^ This goal will require building a diagnostic system that can prepare for and respond to ‘health shocks’, such as outbreaks and natural disasters, and also withstand the chronic stresses that accompany long-term resource constraints.^31^ A systems approach that encompasses both emergency and long-term timeframes needs to be present at the outset of the diagnostic development process, not only at the point at which new technologies are deployed. Most importantly, diagnostic futures need to be designed with the input of the people who work in and use them and they need to incorporate the insight that local experts have gained on the front line of global health innovation.
